# Evaluation of the antidiarrheal activity of the 80% hydromethanolic crude extract and solvent fractions of *Terminalia brownii* Fresen (Combretaceae) leaves in *Swiss Albino* mice

**DOI:** 10.3389/fphar.2024.1510171

**Published:** 2025-01-27

**Authors:** Khalid Ibrahim Kassaw, Dawit Zewdu Wondafrash, Jibril Seid Yesuf, Mestayet Geta Mengistie

**Affiliations:** ^1^ Department of Biomedical Science, School of Medicine, College of Medical and Health Sciences, Samara University, Semera, Ethiopia; ^2^ Department of Pharmacology, School of Medicine, Saint Paul’s Hospital Millennium Medical College, Addis Ababa, Ethiopia; ^3^ Department of Pharmacology, College of Medicine and Health Sciences, University of Gondar, Gondar, Ethiopia

**Keywords:** antidiarrheal activity, *Terminalia brownii* Fresen, Combretaceae, hydromethanolic crude extract, castor oil-induced diarrhea

## Abstract

**Background:**

Although diarrhea is a preventable disease, it continues to have a significant impact on global health, with the burden being much greater in Sub-Saharan Africa. Medicinal plants represent affordable and locally available resources to address many diseases, including diarrhea. Thus, this study aimed to investigate the antidiarrheal activities of the 80% hydromethanolic crude extract and solvent fractions of *Terminalia brownii* Fresen (Combretaceae) leaves in Swiss Albino mice.

**Methods:**

This study was carried out by administering the 80% hydromethanolic crude extract and solvent fractions from the crude extract, including the n-hexane fraction (NHF), ethyl acetate fraction (EAF), and aqueous fraction (AF) at doses of 100 mg/kg, 200 mg/kg, and 400 mg/kg, respectively, to the mice. The effects of these extracts and solvent fractions on reducing/delaying diarrhea were compared with the impact of the solvent used for reconstitution and a standard drug (loperamide 3 mg/kg or atropine 5 mg/kg), as well as with each other.

**Results:**

The hydromethanolic crude extract and ethyl acetate fraction at all tested doses significantly reduced wet defecation (*P* < 0.05). In addition, total defecation was significantly reduced at a dose of 200 mg/kg (*P* < 0.05) and 400 mg/kg (*P* < 0.001) of the crude extract and ethyl acetate fraction at all tested doses (*P* < 0.01. The aqueous fraction at 200 mg/kg and 400 mg/kg and the n-hexane fraction at 400 mg/kg (*P* < 0.05) significantly inhibit wet and total defecation. Likewise, in the enteropooling test, the crude extract and ethyl acetate fractions showed a significant reduction in the weight and volume of intestinal contents at a dose of 200 mg/kg (*P* < 0.05) and 400 mg/kg (*P* < 0.05). Castor oil-induced intestinal motility was significantly reduced (*p* < 0.001) by the crude extract and ethyl acetate fractions at all tested doses.

**Conclusion:**

The result from this study suggests significant antidiarrheal activity of *Terminalia brownii* leaves, which validates its traditional use. The 80% hydromethanolic crude extract and ethyl acetate solvent fractions of *Terminalia brownii* Fresen leaves have shown better antidiarrheal activity.

## Introduction

Diarrhea is a common health problem worldwide, with approximately 2 billion cases of diarrheal disease occurring each year, predominantly affecting children, and resulting in 1.7 billion episodes of childhood diarrhea annually (Collaborators and Ärnlöv, 2020; Black et al., 2019). Diarrhea involves a decrease in absorption, increased fluid secretion, and an increase in the motility of the intestinal tract, resulting in a loss of electrolytes (sodium, chloride, potassium, and bicarbonate) in the liquid stool and, in many cases, dehydration and death (Riddle et al., 2016). In addition to causing death, diarrheal diseases impair normal food intake and nutrient absorption, resulting in malnutrition, which can have long-term consequences such as impaired growth, cognitive development, and increased susceptibility to chronic diseases. Children who are malnourished, on the other hand, have a higher frequency and longer duration of diarrheal illnesses (Guerrant et al., 2013).

Antidiarrheal drugs are grouped into several categories, including antimotility agents, adsorbents, antisecretory compounds, antibiotics, and intestinal microflora. These drugs may have a specific effect, such as when antibiotics are used to treat secretory diarrheas due to enterotoxigenic bacteria, or they may have a non-specific antidiarrheal effect. These non-specific effects may involve pharmacological actions on intestinal transit, intestinal transport, or stool viscosity that result in symptomatic improvement. Usually, these drugs are not curative but palliative (Trevor, 2017; Schiller, 1995; DiPiro et al., 2014).

Despite advances in understanding and management, diarrhea disease remains one of the leading causes of death in developing countries such as Ethiopia (Alebel et al., 2018). Currently available antidiarrheal drugs can help relieve diarrhea symptoms, usually aimed at reducing the discomfort, dehydration, and inconvenience caused by frequent bowel movements. However, their clinical utilities are restricted by side effects, limited indications, and the development of drug resistance by pathogens (Khansari et al., 2013). Oral rehydration solution (ORS) is the cornerstone of management to prevent life-threatening dehydration associated with diarrhea. However, ORS is unable to reduce the volume, frequency, or duration of diarrhea, and it has been reported that ORS is not as widely used as it could be (Ma et al., 2022). Traditional medicinal plants are widely utilized and play a major part in primary healthcare delivery in Ethiopia in both rural and urban regions (Agidew, 2022). Diarrhea is one of the most prominent diseases treated by traditional medicinal plants in Ethiopia (Woldeab et al., 2018). In resource-limited areas like Ethiopia, the WHO (Organization, 2017) has supported studies relevant to the treatment and prevention of diarrheal diseases using traditional medical practices (Organization, 2017).

An ethnobotanical study conducted on the leaves of the *Terminalia brownii* Fresen has shown its claimed use in treating diarrhea, liver disease, and gastric ulcers in different parts of Ethiopia (Kewessa et al., 2015; Belayneh and Bussa, 2014; Degu et al., 2022). Previous ethnopharmacological investigations conducted on the *Terminalia brownii* Fresen plant leaf extract have demonstrated different pharmacologic activities, which could be the reason for its claimed antidiarrheal activity. The extract of *Terminalia brownii* Fresen leaves showed antibacterial activity against most microbes causing diarrhea (Mechesso et al., 2016). Additionally, methanol extracts from the leaves of this plant also revealed radical scavenging activity (Teshome et al., 2015), analgesic activity (Periasamy et al., 2015), and antioxidant properties (Sintayehu et al., 2017), suggesting that the claimed antidiarrheal activity of the extract could be attributed to the inhibition of castor oil-induced prostaglandin synthesis. Furthermore, diarrhea is believed to impair the intestinal antioxidant defense system and induce other oxidative stress disorders. The antioxidant activity and radical scavenging activities of the extract may be responsible for its claimed antidiarrheal action (Ma et al., 2022).

For the reasons listed above, the present study aimed to evaluate the antidiarrheal activity of the leaves of *Terminalia brownii* Fresen extracts using different diarrheal model and determine whether the traditional medicinal use has a scientifically justified basis, as well as to provide an alternative source for developing new antidiarrheal drugs.

## Materials and methods

### Chemicals and reagents

Castor oil, (Amman Pharmaceutical Industries, Jordan), loperamide (Medochemie Ltd., Cyprus), distilled water (University of Gondar Teaching Specialized Hospital, Ethiopia), methanol (Alpha Chemika, India), activated charcoal (Acuro Organics Ltd., New Delhi), n-hexane (Central Drug House Ltd. India), ethyl acetate (Central Drug House Ltd. India), atropine sulfate (Humanwell Pharmaceutical PLC, Ethiopia), chloroform (CARLO ERBA, Reagents, SAS, France), Tween 80 (Atlas Chemical Industries, India), sulfuric acid (HiMedia Laboratories Pvt. Ltd.), hydrochloric acid (Blulux Laboratories Ltd., India), ferric chloride (HiMedia Laboratories Pvt. Ltd.) glacial acetic acid (Alpha Chemika, India), Mayer’s reagents (Research Lab Fine Chem Industries, India), Wagner’s reagents (Research Lab Fine Chem Industries, India), and sodium hydroxide (Qualichem Laboratories Ltd., India).

### Plant material

Fresh leaves of *Terminalia brownii* Fresen were collected from nearby Hara Town, around farmland in the Guba Lafto District, Amhara National Regional State, 425 km from the city of Gondar on 03 January 2023. They were transported by wrapping them in a plastic bag. The leaves were identified and authenticated by the botanist at the University of Gondar, Mr. Getnet Chekole. A voucher specimen (number 01/KI/2023) was deposited in the herbarium of the Department of Biology, University of Gondar, for future reference.

### Experimental animals

Healthy Swiss Albino mice of either sex, weighing 25–30 gm and aged 8–12 weeks, were used. The animals were obtained from the animal house of the Department of Pharmacology, School of Pharmacy, University of Gondar. All animals were housed in plastic cages at room temperature in an air-conditioned room with a 12-hour light/dark cycle and had access to pellet diet and clean water *ad libitum* throughout the experimental period. Before starting any experiment, animals were allowed a 1-week acclimatization period to the experimental environment, and all the experiments were carried out according to the internationally accepted laboratory animal care and use guidelines (Care IoLARCo and Animals UoL, 1986). The protocol was approved by the Ethics Review Committee of the Department of Pharmacology with reference number (SOP4/50/2015).

### Plant extraction

The leaves of *Terminalia brownii* Fresen were rigorously washed with tap water, dried in the shade, and extracted.

#### Hydromethanolic crude extraction (80% methanol), MeOH

One thousand grams of powdered leaves were measured and acquired using a digital balance (EPH 00 Abron Exports). The 1,000 g powder was cold macerated in three equal-sized Erlenmeyer conical flasks, with a total of 6 L of 80% hydromethanol (1:6). It was left for 3 days with occasional shaking before being filtered through gauze and then Whatman No. 1 filter paper on day 4. The residue in each macerating flask was remacerated twice with 1 L of 80% hydromethanol in each Erlenmeyer conical flask, for a total of 3 L (1:3). The filtrates, on the other hand, were combined and concentrated using a rotary evaporator (Yamato Scientific Co. Ltd., Japan) set at 40°C, after which the concentrate was frozen in a deep freezer overnight. The water content of the frozen filtrate was then removed by lyophilization within a vacuum freeze dryer (Lab Freeze Instruments Group, Ltd., Germany). Finally, the dried filtrate was well packed in an air-tight container and refrigerated at 4°C until use.

#### Solvent fractions

Solvent fractionation was carried out after taking 60 g of the 80% hydromethanolic crude extract from the leaves. N-hexane, ethyl acetate, and distilled water were the solvents used for fraction preparation. The crude extract was first dissolved in a 1,000 mL glass beaker with 250 mL of distilled water, then an equivalent volume of n-hexane was added, and then the bulk solution was transferred to a separating funnel and stirred occasionally for a few hours until a clear layer appeared. The upper layer (n-hexane) was filtered. The residue was remacerated twice more with the same volume of n-hexane and in the same manner as the described above. The filtrate fractions were then mixed to form the n-hexane fraction (NHF) and subjected to evaporation using a rotary evaporator (Yamato Scientific Co. Ltd., Japan) set at 40°C, and then the filtrate was placed in an oven at 40°C for 1 week to remove the remaining solvent. The second solvent (250 mL), ethyl acetate, was added to the separating funnel containing aqueous residue and repeated in the same manner as n-hexane. The ethyl acetate layer was then separated from the residue, which was the upper portion of the mixture, and used as the ethyl acetate fraction (EAF). It was then subjected to evaporation and placed in an oven at 40°C similar to n-hexane. The final residue was used as an aqueous fraction (AF) (Zayede et al., 2020). The AF was lyophilized to remove water; after drying, the solvent fractions were stored in an air-tight container in a refrigerator until further use for the evaluation of the antidiarrheal activity and phytochemical constituent. The yield of both the crude extract and solvent fraction was calculated according to the following formula.

$$\%\text{ yield}=\frac{\text{weight}\;\text{of}\;\text{the}\;\text{extract}}{\text{weight}\;\text{of}\;\text{the}\;\text{plant}\;\text{material}}*100.$$

### Acute oral toxicity

An acute oral toxicity test was performed based on the Organization for Economic Cooperation and Development guideline number 425. The limit test was performed using five healthy, non-pregnant, nulliparous female Swiss Albino mice as per the protocol. The mice were kept for a week before dosing to acclimatize to the environment and then fasted for 4 h from food but had normal access to water. The doses were calculated according to the fasted body weight. Primarily, one mouse was given the limit dose of 2,000 mg/kg via oral gavage, and food was withheld for a further 2 h after administration. Based on the outcome of the first animal in the 24-hour follow-up, four additional animals were sequentially dosed at 2,000 mg/kg so that a total of five animals were tested. Animals were observed continuously during the first 30 min after dosing, periodically during the first 24 h (with special attention given during the first 4 h), and daily thereafter, for a total of 14 days.

### Grouping and dosing of animals

In all three experimental models, Swiss Albino mice were randomly assigned into five groups, each group consisting of six mice. Group I (negative control) mice were given the vehicle at a volume of 10 mL/kg body weight (distilled water for the control group in the 80% hydromethanolic crude extract and aqueous fraction test or 2% Tween 80 in distilled water for the control group in the n-hexane and ethyl acetate fraction test). Group II served as the positive control and was treated with standard drugs (loperamide for castor oil-induced diarrhea and enteropooling and atropine sulfate for the gastrointestinal motility test), and depending on the results of the acute toxicity study, three different doses (100, 200, and 400 mg/kg) of the 80% hydromethanolic extract and solvent fractions of *Terminalia brownii* Fresen were tested orally on groups III, IV, and V of mice, respectively. The middle dose (200 mg/kg) is one-tenth of the dose used during the acute toxicity study; the low dose (100 mg/kg) is half of the middle dose; and the high dose (400 mg/kg) is twice the middle dose, based on OECD guidelines (No, 2008).

### Determination of antidiarrheal activity

#### Castor oil-induced diarrhea

The castor oil-induced diarrhea model was carried out as per the method described by Umer et al. (2013). In this experiment, 30 Swiss Albino mice were fasted for 18 h and divided into five groups, with six animals in each group. After 1 h of administration of the respective treatment dosages, as stated in the grouping and dosing section, the mice were given 0.5 mL of castor oil and housed separately in distinct plastic cages, where the floor was lined with transparent paper. The paper was changed every hour for a total of 4 h. During a 4-hour observation period, the onset of diarrhea, frequency of defecation (wet and total), and the weight of fecal output (wet and total feces in grams) were recorded for each mouse. The percentage of diarrheal inhibition and percentage inhibition of defecation were determined according to the following formulas (Tadesse et al., 2014).

$$\%\text{ inhibition of diarrhea}=\frac{\text{Mean}\;\text{number}\;\text{of}\;\text{wet}\;\text{defecation}\;\left(\text{negative}\;\text{control}-\text{test}\right)}{\text{Mean}\;\text{number}\;\text{of}\;\text{wet}\;\text{defecation}\;\text{of}\;\text{negative}\;\text{control}}*100,$$

$$\%\text{ inhibition of defeciation}=\frac{\text{Total}\;\text{number}\;\text{of}\;\text{feces}\;\text{in}\;\text{the}\;\left(\text{negative}\;\text{control}-\text{treated}\;\text{group}\right)}{\text{Total}\;\text{number}\;\text{of}\;\text{feces}\;\text{in}\;\text{the}\;\text{negative}\;\text{control}}*100.$$

#### Castor oil-induced enteropooling

The method followed by Sisay et al. (2017) was used in this study. Thirty mice, divided into five groups, were deprived of both food and water for 18 h and treated as stated in the grouping and dosage section. After 1 h of treatment, 0.5 mL of castor oil was administered, and animals were euthanized by cervical dislocation 1 h following castor oil administration. The abdomen of each animal was then opened; the small intestine was ligated at both the pyloric sphincter and the ileocecal junction and carefully dissected out. Each small intestine was weighed on an electrical balance, after which the contents of the intestine were milked into a graduated tube, and the volume of the fluid was recorded. The empty intestine was then reweighed, and the difference between full and empty intestines was calculated. The percentage of reduction in intestinal contents in terms of volume and weight was estimated relative to the negative control using the following formulas.

$$\text{Mean }p\text{ercentage volume inhibition}=\frac{\text{MVICC}-\text{MVICT}}{\text{MVICC}}*100,$$

where MVICC is the mean volume of the intestinal content of the control group and MVICT is the mean volume of the intestinal content of the test group.

$$\text{Mean }p\text{ercentage weight inhibition}=\frac{\text{MWICC}\;–\text{MWICT}}{\text{MWICC}}*100,$$

where MWICC is the mean weight of the intestinal content of the control group and MWICT is the mean weight of the intestinal content of the test group.

#### Castor oil-induced gastrointestinal motility

The gastrointestinal motility test was evaluated according to the method described by Tadesse et al. (2017). Thirty Swiss albino mice of either sex were fasted for 18 h with free access to water, and later, they were divided and treated as described earlier. An hour later, 0.5 mL of castor oil was administered via oral gavage, followed by the administration of 1 mL of 5% charcoal suspension in distilled water 1 h after castor oil treatment. The animals were then euthanized by cervical dislocation 30 min after a charcoal meal, and the small intestine was dissected out from pylorus to cecum. The distance traveled by the marker and the total length of the intestine were then measured. The peristaltic index and percentage of inhibition were calculated using the following formulas.

$$\text{Peristaltic index }\left(\text{PI}\right)=\frac{\text{Mean}\;\text{distance}\;\text{travelled}\;\text{by}\;\text{charcoal}\;\text{meal}}{\text{Mean}\;\text{length}\;\text{of}\;\text{small}\;\text{intestine}}*100,$$

$$\%\text{ of inhibition}=\frac{\text{PIC}-\text{PIT}}{\text{PIC}}*100,$$

where PIC is the peristaltic index of the control and PIT is the peristaltic index of the test group.

#### *In vivo* antidiarrheal index

The *in vivo* antidiarrheal index (ADI) (Sintayehu et al., 2017) of the 80% hydromethanolic crude extract, solvent fraction, and standard drug was determined according to the following formula developed by Than et al. (1989):

$$\text{ADI}=\sqrt[{3}]{Dfreq\;X\;Gmeq\;X\;Pfreq,}$$

where Dfreq is the delay in defecation time as a percentage of the negative control, Gmeq is the gut meal travel reduction as a percentage of the negative control, and Pfreq is the reduction in purging frequency in the number of wet stools as a percentage of the negative control.

$$\text{Dfreq}=\frac{\text{Mean}\;\text{onset}\;\text{of}\;\text{diarrhea}\;\text{in}\;\text{test}\;\text{group}-\text{Mean}\;\text{onset}\;\text{of}\;\text{diarrhea}\;\text{in}\;\text{control}\;\text{group}}{\text{Mean}\;\text{onset}\;\text{of}\;\text{diarrhea}\;\text{in}\;\text{control}\;\text{group}\;}*100.$$

### Preliminary phytochemical screening

The qualitative phytochemical investigations of the hydromethanolic crude extract and solvent fractions of *Terminalia brownii* Fresen leaves were conducted according to standard chemical tests reported elsewhere (Degu et al., 2016).

### Ethical clearance

After the approval of the proposal by the Graduate Committee of the Department of Pharmacology, an ethical approval letter was obtained from the Department of Pharmacology with a reference number (SOP4/50/2015).

### Statistical analysis

The results of the experiment were expressed as the mean ± standard error of mean, and comparisons were made between the negative control, positive control, and treatment groups of various doses using one-way analysis of variance (ANOVA), followed by Tukey’s post-hoc test with statistical package for the social sciences (SPSS) version 26. All data were analyzed at a 95% confidence interval, and P-values less than 0.05 were considered statistically significant.

## Results

### Acute toxicity study

In the oral acute toxicity test using the limit dose of 2,000 mg/kg body weight, the 80% hydromethanolic leaf extract of *Terminalia brownii* Fresen was found to be safe as there was no mortality or signs of overt toxicity noted in the 14-day observation of the mice.

### Phytochemical test

The MeOH crude extracts and solvent fractions of *Terminalia Brownii* Fresen leaves were screened for the possible presence or absence of phyto-constituents (Table 1). Alkaloids and anthraquinones were not detected in both crude extract and solvent fractions. Tannins, saponins, flavonoids, terpenoids, phenols, and glycosides were present in the crude extract and solvent fractions. The EAF concentrated most of the possible presence of secondary metabolites.

**TABLE 1:** Preliminary phytochemical screening results of the 80% hydromethanolic extract and solvent fractions of *Terminalia brownii* Fresen leaves.

Secondary metabolites	Method used for screening	MeOH-E	NHF	EAF	AF
Flavonoids	Alkaline reagent test	+	+	+	+
Terpenoids	Salkowski test	+	+	+	-
Saponins	Foam test	+	-	-	+
Tannins	Ferric chloride test	+	-	+	+
Phenols	Ferric chloride test	+	-	+	-
Alkaloids	Mayer test	-	-	-	-
Cardiac glycosides	Keller–Kiliani test	+	-	+	+
Steroids	Salkowski test	+	+	+	-
Anthraquinones	Conc. HCl test	-	-	-	-

MeOH-E, hydromethanolic extract; NHF, n-hexane fraction; EAF, ethyl acetate fraction; AF, aqueous fraction; +, present; -, absent.

### Antidiarrheal activity

#### Effects on castor oil-induced diarrhea in mice

The 80% hydromethanolic crude extract of the leaves of *Terminalia brownii* Fresen exhibits antidiarrheal activity by significantly delaying the onset of diarrhea at doses of 200 mg/kg and 400 mg/kg. It was also found to significantly reduce the number of wet defecations, weight of wet feces at all tested doses, frequency of total defecation, and weight of total feces at 200 mg/kg and 400 mg/kg doses of the extract. However, no apparent difference was observed for MeOH (100 mg/kg) in reducing the frequency and weight of wet faces and prolongation in diarrhea onset compared with the negative control (Table 2).

**TABLE 2:** Antidiarrheal effects of the 80% hydromethanolic crude extract and solvent fractions of *Terminalia brownii* Fresen leaves on castor oil-induced diarrhea model in mice.

Extract	Dose mg/kg	Onset of diarrhea (min)	No of wet feces	Total number of feces	Average weight of wet feces (gm)	Average weight of total feces (gm)	Inhibition of diarrhea (%)	Inhibition of defecation (%)
80% MeOH-E	Control	48.83 ± 2.62	9.50 ± 0.76	10.66 ± 0.76	0.83 ± 0.10	0.92 ± 0.06	—	—
	LOP 3	111.83 ± 8.05^a3c2^	2.33 ± 0.21^a3c2d1^	3.83 ± 0.47^a3c2d1^	0.20 ± 0.06^a3^	0.29 ± 0.06^a2c1^	75.43	64.07
	MeOH 100	61.83 ± 2.54^b2d1e2^	5.50 ± 0.50^a3b2^	8.83 ± 0.65^b2e1^	0.45 ± 0.03^a1^	0.69 ± 0.12^b1^	42.10	17.16
	MeOH 200	100.66 ± 12.92^a2c1^	4.66 ± 0.49^a3b1^	7.16 ± 0.79^a1b1^	0.34 ± 0.03^a2^	0.43 ± 0.05^a2^	50.87	32.83
	MeOH 400	106.33 ± 10.97^a2c2^	3.33 ± 0.66^a3^	5.16 ± 1.01^a3c1^	0.31 ± 0.11^a2^	0.38 ± 0.13^a2^	64.91	51.59
Solvent fraction	Control	42.50 ± 6.26	9.16 ± 0.54	10.83 ± 0.30	0.88 ± 0.04	0.98 ± 0.06	—	—
	Lop 3	105.66 ± 17.70^a3c2d2e2^	2.50 ± 0.42^a3c2d2^	3.50 ± 0.42^a1c2d1^	0.25 ± 0.04^a3c3d3^	0.28 ± 0.05^a3c2d2^	72.70	67.68
	NHF 100	51.00 ± 3.26^b2^	7.33 ± 0.71^b2^	8.33 ± 0.95^b2^	0.68 ± 0.06^b3e1^	0.74 ± 0.07^b2^	19.97	23.08
	NHF 200	55.50 ± 3.25^b2^	7.00 ± 1.00^b2^	8.00 ± 1.18^b2^	0.65 ± 0.06^b3^	0.73 ± 0.07^b2^	23.58	26.13
	NHF 400	58.16 ± 5.06^b2^	5.33 ± 0.88^a1^	7.00 ± 1.18^a1^	0.42 ± 0.05^a3c1^	0.53 ± 0.07^a2^	41.81	35.36
	Control	42.50 ± 6.26	9.16 ± 0.54	10.83 ± 0.30	0.88 ± 0.04	0.98 ± 0.06	—	—
	Lop 3	105.66 ± 17.70^a2c1^	2.50 ± 0.42^a3c2^	3.50 ± 0.42^a3c3^	0.25 ± 0.04^a3c2^	0.28 ± 0.05^a3c3d1^	72.70	67.68
	EAF 100	61.33 ± 7.18^b1e1^	6.33 ± 0.84^a1b2^	7.66 ± 0.61^a2b3e2^	0.60 ± 0.05^a1b2e2^	0.68 ± 0.03^a2b3e2^	30.89	29.27
	EAF 200	88.83 ± 8.10^a1^	4.50 ± 0.61^a3^	5.50 ± 0.61^a3^	0.47 ± 0.05^a3^	0.53 ± 0.05^a3b1^	50.87	49.21
	EAF 400	104.50 ± 4.63^a2c1^	3.66 ± 0.76^a3^	4.16 ± 0.83^a3c2^	0.35 ± 0.06^a3^	0.37 ± 0.07^a3c2^	60.04	61.58
	Control	46.00 ± 4.39	9.33 ± 0.84	10.50 ± 0.76	0.85 ± 0.13	0.91 ± 0.13	—	—
	LOP 3	116.16 ± 10.85^a3c3d2^	2.33 ± 0.42^a3c3^	3.33 ± 0.33^a3c2^	0.23 ± 0.02^a3c2d1^	0.28 ± 0.07^a3c2d1^	75.02	68.28
	AF 100	58.16 ± 6.26^b3e1^	7.00 ± 0.89^b3e1^	8.66 ± 1.25^b2^	0.70 ± 0.05^b2^	0.76 ± 0.08^b2^	24.97	17.52
	AF 200	72.33 ± 7.50^b2^	4.83 ± 0.60^a3^	5.66 ± 0.66^a2^	0.58 ± 0.05^b1^	0.62 ± 0.05^b1^	48.23	46.09
	AF 400	91.83 ± 7.37^a2c1^	4.16 ± 0.70^a3c1^	5.50 ± 0.84^a2^	0.49 ± 0.07^a1^	0.57 ± 0.06^a1^	55.41	47.61

Data are presented as the mean ± SEM (n = 6); analysis was performed using one-way ANOVA, followed by Tuckey *post-hoc* test; ^a^ compared with negative control values; ^b^ compared with loperamide; ^c^ compared with 100 mg/kg; ^d^ compared with 200 mg/kg; ^e^ compared with 400 mg/kg; 1 *p* < 0.05, 2 *p* < 0.01, and 3 *p* < 0.001; MeOH, hydromethanolic extract; LOP 3, loperamide 3 mg/kg; NHF, n-hexane fraction; EAF, ethyl acetate fraction; AF, aqueous fraction. Control; administered with 10 mL/kg of distilled water (for the crude extract and aqueous fraction) and 10 mL/kg of 2% Tween 80 (for n-hexane fraction and ethyl acetate fraction).

Among these solvent fractions, the EAF significantly reduced wet defecation and total defecation at 100 mg/kg (*p* < 0.05), 200 mg/kg (*p* < 0.001), and 400 mg/kg (*p* < 0.001). The percentage of diarrheal inhibition obtained from the EAF test compared to the negative control was 30.89%, 50.87%, and 60.04% at 100 mg/kg, 200 mg/kg, and 400 mg/kg doses of the fraction, respectively. The EAF also showed a significant reduction in the weight of both wet and total fecal output at 100 mg/kg (*p* < 0.05), 200 mg/kg (*p* < 0.001), and 400 mg/kg (*p* < 0.001). The onsets of diarrhea were also prolonged significantly at doses of 200 mg/kg (*p* < 0.05) and 400 mg/kg (*p* < 0.01) but not at 100 mg/kg dose of the fraction.

#### Effects on castor oil-induced enteropooling

As shown in Table 4, in this model, administration of the crude extract and solvent fraction resulted in a significant reduction in both the volume of the intestinal content and the weight of intestinal content compared to the negative control. The hydromethanolic crude extract of *Terminalia brownii* leaves at the dose of 200 mg/kg (*p* < 0.05) and 400 mg/kg (*p* < 0.01) significantly reduced the volume of fluid accumulation in the intestine, with percentage inhibitions of 37.87% and 45.45%, respectively, compared with the negative control.

Regarding solvent fractions, the EAF significantly reduced intestinal volume accumulation and weight of intestinal contents at 200 mg/kg (*p*-value <0.05) and 400 mg/kg (*p*-value <0.05). The highest inhibition of both intestinal volume and weight of intestinal content was observed at 400 mg/kg of the EAF (43.79% and 42.28%, respectively).

#### Effects on castor oil-induced intestinal motility in mice

In this model, the crude extract at all tested doses retarded the intestinal motility of charcoal meal relative to the negative control group, and the results were statistically significant (*p* < 0.001). The percentage reduction in intestinal motility was 37.16%, 49.20%, and 53.06% at the doses of 100, 200, and 400 mg/kg of the extract, respectively, while the standard drug atropine produced significant inhibition of 70.47% (*p* < 0.01) in intestinal motility compared with the negative control. Regarding the activity of solvent fractions, the EAF showed a significant reduction of intestinal transit of the charcoal meal at all tested doses relative to the negative control (*p* < 0.001) with percentage inhibitions of 46.13%, 53.25%, and 59.29% at 100 mg/kg, 200 mg/kg, and 400 mg/kg, respectively.

#### *In vivo* antidiarrheal index

To demonstrate the combined effect of the models discussed above, the *in vivo* antidiarrheal index of the crude leaf extract and solvent fraction of *Terminalia brownii* Fresen was computed by combining three parameters, namely, percentage delay in defecation (time of onset, Dfreq), percentage gut meal travel distance (Gmeq), and percentage purging frequency in the number of wet stools (Pfreq) correction; As shown in Table 5, 34.66%, 64.28%, and 74.01% were recorded as *in vivo* ADI for 100, 200, and 400 mg/kg doses of hydromethanolic crude extract, respectively. The highest *in vivo* antidiarrheal index was observed by the standard drug (88.18%).

From solvent fractions, the maximum dose of each fraction showed the highest antidiarrheal index. The EAF showed ADI values of 39.81%, 66.59%, and 80.37% for 100 mg/kg, 200 mg/kg, and 400 mg/kg, respectively. The AF showed ADI values of 21.38%, 41.93%, and 55.24% for 100 mg/kg, 200 mg/kg, and 400 mg/kg, respectively, and NHF showed 12.55%, 24.00%, and 36.49% for 100 mg/kg, 200 mg/kg, and 400 mg/kg, respectively.

## Discussion

The present study aimed to evaluate the antidiarrheal activity of the 80% MeOH-E and solvent fractions of *Terminalia brownii* Fresen leaves using different experimental models of diarrhea in mice. In all models, diarrhea was induced by administering castor oil to each mouse (Oghenesuvwe et al., 2018). In the first castor oil-induced diarrhea model, MeOH-E has shown a significant effect on all parameters measured, as evidenced by a significant prolongation of the onset of diarrhea (200 mg/kg and 400 mg/kg), a significant reduction of wet defecation at all tested doses, and total defecation (200 and 400 mg/kg) compared to the negative control. In addition, the crude extract showed a significant decrement in the weight of wet feces at all tested doses. The two higher doses of the extract (200 mg/kg and 400 mg/kg) also significantly reduced the weight of total feces compared to the negative control. However, the lower dose (100 mg/kg) showed no significant reduction in the weight of total feces and prolongation of the onset of diarrhea. The results of this investigation suggested that wet defecation was more effectively suppressed when the test extract dose was increased, which could be related to the test extract’s antisecretory and antimotility properties (Mekonnen et al., 2018).

Regarding the solvent fractions, the EAF showed the utmost activity in most of the parameters of this model (except the onset at the lower dose) relative to the negative control, followed by the AF, which significantly reduced both wet and total defecations at the medium and higher doses of the fractions and significantly reduced the weight of wet and total feces and the prolongation of the diarrhea-free period at 400 mg/kg.

The inhibition of the assessed diarrheal parameters (onset of diarrhea, wet defecation, total defecation, and weight of feces) might have been because of active medium polar and polar secondary metabolites, which are attracted to the EAF, MeOH-E, and AF both in quality and quantity (Zayede et al., 2020). This might explain the impact of the crude extract, EAF at all doses, and AF at 200 mg/kg and 400 mg/kg on delaying or reducing diarrhea. The lack of NHF’s activity at all three doses in delaying the onset of diarrhea and its minimal activity on other parameters in this model may be attributed to the presence of less active non-polar secondary metabolites (Mengistu et al., 2015).

The study further investigated anti-enteropooling effect of the plant using castor oil-induced enteropooling to explore any potential antisecretory activity (Whyte and Jenkins, 2012). As shown in Table 1, both intestinal volume and weight of intestinal content were significantly inhibited at 200 mg/kg and 400 mg/kg of the hydromethanolic crude extract compared to the negative control, respectively. Among solvent fractions, the EAF showed significant activity in both reducing intestinal volume and weight of intestinal contents at the middle and higher dose levels, while both the NHF and AF showed significant activity at higher doses (400 mg/kg). The pronounced inhibition of castor oil-induced intestinal fluid accumulation in the EAF could be related to the presence of many active phytochemical constituents, as shown in Table 3, both in quality and quantity in the EAF, which may work individually and synergistically to inhibit diarrhea (Ayalew et al., 2022).

**TABLE 3:** Effects of the 80% hydromethanolic crude extract and solvent fractions of *Terminalia brownii* Fresen leaves on gastrointestinal fluid accumulation in mice.

Extract	Dose mg/kg	Volume of intestinal contents (Alebel et al., 2018)	% inhibition	Weight of intestinal contents (gm)	% inhibition
80% MeOH-E	Control	0.66 ± 0.04	—	0.76 ± 0.03	—
	LOP 3	0.21 ± 0.04^a3c2^	68.18	0.27 ± 0.06^a3c2^	64.47
	MeOH 100	0.50 ± 0.05^b2^	24.24	0.60 ± 0.06^b2^	21.05
	MeOH 200	0.41 ± 0.05^a1^	37.87	0.50 ± 0.07^a1^	34.21
	MeOH 400	0.36 ± 0.05^a2^	45.45	0.47 ± 0.05^a1^	38.15
Solvent fraction	Control	0.71 ± 0.06	—	0.83 ± 0.07	—
	Lop 3	0.20 ± 0.03^a3c3d2^	71.83	0.29 ± 0.06^a3c2d1^	65.06
	NHF 100	0.65 ± 0.08^b3^	8.4	0.73 ± 0.09^b2^	12
	NHF 200	0.54 ± 0.07^b2^	23.94	0.64 ± 0.05^b1^	22.89
	NHF 400	0.43 ± 0.04^a1^	39.43	0.50 ± 0.03^a1^	39.75
	Control	0.71 ± 0.06	—	0.83 ± 0.07	—
	Lop 3	0.20 ± 0.03^a3c1^	71.83	0.29 ± 0.06^a3c2^	65.06
	EAF 100	0.53 ± 0.07^b1^	25.35	0.67 ± 0.05^b2^	18.83
	EAF 200	0.41 ± 0.04^a1^	41.45	0.51 ± 0.05^a1^	38.47
	EAF 400	0.40 ± 0.09^a1^	43.79	0.48 ± 0.09^a1^	42.28
	Control	0.68 ± 0.08	—	0.80 ± 0.08	—
	LOP 3	0.16 ± 0.03^a3c3d1^	75.60	0.28 ± 0.07^a3c2d1^	64.37
	AF 100	0.61 ± 0.07^b3^	9.75	0.72 ± 0.08^b2^	10
	AF 200	0.45 ± 0.06^b1^	34.14	0.62 ± 0.06^b1^	23.02
	AF 400	0.40 ± 0.04^a1^	41.46	0.49 ± 0.05^a1^	38.12

Data are presented as the mean ± SEM (n = 6); analysis was performed using one-way ANOVA, followed by Tuckey *post-hoc* test; ^a^compared with negative control; ^b^compared with loperamide; ^c^compared with 100 mg/kg; ^d^compared with 200 mg/kg; ^e^compared with 400 mg/kg; ^1^ *p*<0.05, ^2^ *p* < 0.01, and ^3^ *p* < 0.001; MeOH, methanolic extract; LOP 3, loperamide 3 mg/kg; NHF, n-hexane fraction; EAF, ethyl acetate fraction; AF, aqueous fraction; control; administered with 10 mL/kg of distilled water (for the crude extract and aqueous fraction) and 10 mL/kg of 2% Tween 80 (for n-hexane fraction and ethyl acetate fraction).

The result from the enteropooling model indicates that the antidiarrheal activity of the current plant extract may work by inhibiting intestinal secretion produced by ricinoleic acid, which causes irritation and inflammation of the intestinal mucosa, leading to prostaglandin release, which, in turn, provokes the stimulation of secretion, thereby preventing the reabsorption of sodium chloride and water (Pierce et al., 1971).

Phytochemical constituents revealed in the current plant may be related to the extract’s anti-enteropooling activity. Nitric oxide production is known to be decreased by the presence of phytochemicals such as flavonoids, terpenoids, and steroids (Adela et al., 2022). Cystic fibrosis transmembrane conductance regulator (CFTR) protein, which transports chloride ions from epithelial cells to the lumen, thereby increasing secretion, is significantly upregulated by castor oil (Guo et al., 2022). On the other hand, tannins present in the current plant extract inhibit CFTR or calcium-activated channels, thereby decreasing secretion in the small intestine and colon (Soares et al., 2020). Terpenoids have anti-inflammatory properties, which may be attributed to their ability to inhibit the production of prostaglandin E2 (Prakash, 2017). Phenols have antioxidant activity by neutralizing free radicals, which may involve the inhibition of inflammation, thereby reducing secretion (Alemu et al., 2022).

The third model used to investigate the antidiarrheal activity of *Terminalia brownii* Fresen was castor oil-induced gastrointestinal motility using a charcoal meal test (Sunil et al., 2019). As shown in Table 4, the charcoal meal transited much of the total length of the small intestine (85.43%), indicating an increased level of peristalsis in the negative control group. The hydromethanolic crude extract at all tested doses retarded the intestinal transit of the charcoal meal (*p* < 0.001); the highest percentage inhibition by extracts was seen at 400 mg/kg, and the standard drug produced 70.47% percentage inhibition, which is higher than the test extracts.

**TABLE 4:** Effects of the 80% methanolic crude extract and solvent fractions of *Terminalia brownii* Fresen leaves on gastrointestinal motility in mice.

Extract	Dose mg/kg	Total length of the small intestine (cm)	Distance covered by the charcoal meal (cm)	Peristaltic index (%)	% inhibition
80% MeOH-E	Control	54.50 ± 1.05	46.50 ± 1.17	85.43 ± 2.43	—
	Atro 5	54.16 ± 0.98	13.66 ± 0.80^a3c3d2e1^	25.22 ± 1.33^a3c3d2e1^	70.47
	MeOH 100	53.33 ± 1.14	28.66 ± 1.52^a3b3^	53.68 ± 2.36^a3b3e1^	37.16
	MeOH 200	54.50 ± 1.17	25.00 ± 2.80^a3b2^	45.96 ± 5.28^a3b2^	49.20
	MeOH 400	54.75 ± 2.03	22.00 ± 1.89^a3b1^	40.07 ± 2.90^a3b1c1^	53.06
Solvent fraction	Control	52.66 ± 1.33	42.50 ± 3.38	80.63 ± 6.03	—
	Atro 5	55.50 ± 0.95	14.50 ± 0.99^a3c3d3e3^	26.15 ± 1.80^a3c3d3e2^	67.65
	NHF 100	53.83 ± 2.52	41.00 ± 1.48^b3e1^	76.63 ± 3.09^b3e1^	4.96
	NHF 200	54.50 ± 1.31	35.33 ± 2.69^b2^	65.16 ± 5.64^b3^	19.18
	NHF 400	56.66 ± 1.92	31.00 ± 0.68^a2b3c1^	55.19 ± 3.08^a2b2c1^	31.55
	Control	52.66 ± 1.33	42.50 ± 3.38	80.63 ± 6.03	—
	Atro 5	55.50 ± 0.95	14.50 ± 0.99^a3^	26.15 ± 1.80^a3^	67.56
	EAF 100	53.00 ± 1.00	23.00 ± 2.81^a3^	43.43 ± 5.32^a3^	46.13
	EAF 200	51.50 ± 0.88	19.33 ± 1.85^a3^	37.69 ± 3.88^a3^	53.25
	EAF 400	56.00 ± 1.00	18.16 ± 2.44^a3^	32.82 ± 4.91^a3^	59.29
	Control	51.16 ± 1.62	42.83 ± 1.77	83.75 ± 2.33	—
	Atro 5	54.00 ± 2.06	14.33 ± 0.61^a3c3d3e3^	26.74 ± 1.56^a3c3d3e3^	68.07
	AF 100	50.33 ± 1.40	35.83 ± 2.08^b3^	71.34 ± 4.23^b3e1^	14.81
	AF 200	54.00 ± 1.34	33.16 ± 2.21^a2b3^	61.37 ± 3.59^a3b3^	26.72
	AF 400	53.16 ± 1.88	30.66 ± 1.33^a3b3^	57.79 ± 2.33^a3b3e1^	30.99

Data are presented as the mean ± SEM (n = 6); analysis was performed using one-way ANOVA, followed by Tuckey *post-hoc* test; ^a^compared with negative control values; ^b^compared with loperamide; ^c^compared with 100 mg/kg; ^d^compared with 200 mg/kg; ^e^compared with 400 mg/kg; ^1^ *p* < 0.05, ^2^ *p* < 0.01, and ^3^ *p* < 0.001; MeOH, methanolic extract; Atro 5, atropine 5 mg/kg; NHF, n-hexane fraction; EAF, ethyl acetate fraction; AF, aqueous fraction; control; administered with 10 mL/kg of distilled water (for the crude extract and aqueous fraction) and 10 mL/kg of 2% Tween 80 (for n-hexane fraction and ethyl acetate fraction).

Among solvent fractions, the EAF showed significant activity at all tested doses in reducing intestinal motility and peristalsis index relative to the negative control. It was also shown that the AF at doses of 200 mg/kg and 400 mg/kg significantly reduced gastrointestinal distance traveled by the charcoal meal in mice compared with the negative control, whereas the NHF showed a significant difference only at the higher dose of 400 mg/kg (*p* < 0.05). This could be due to the lack of active phytochemicals in the NHF, such as tannins and phenols, and the possible presence of terpenoids and flavonoids at 400 mg/kg, whereas the pronounced effect in the EAF and AF could be due to the presence of many active phytochemicals, such as flavonoids, tannins, phenols, terpenoids, steroids, and saponins, which might work individually or synergistically (Mekonnen et al., 2018).

The standard drug atropine has an antispasmodic response by inhibiting the cholinergic effect, which promotes sodium chloride and fluid secretion, as well as gastrointestinal motility (Salako et al., 2015). Thus, the current extract could have resulted from its antimotility activity by the atropine-like effect. Another way in which the extract might have reduced motility is by the stimulation of opioid receptors, which inhibits the release of acetylcholine like loperamide (Gerring, 1989). The stimulation of the intestine by alpha-two adrenergic agents such as enkephalins and somatostatins and agents that antagonize the action of prostaglandins in the intestine results in the reduction of peristaltic activity and increased gastrointestinal absorption, which could be other possible mechanisms of the current plant extract (Teferi et al., 2019).

The antidiarrheal index is a method for calculating the cumulative effects of antidiarrheal parameters such as decreased intestinal motility, the delayed onset of defecation, and a reduction in the number of wet stools (Sisay et al., 2017) The higher the ADI value, the greater the effectiveness of the extract in the treatment of diarrhea (Prasad et al., 2014). As shown in Table 5, ADI values were increased with higher doses of the extracts and fractions. The highest ADI value was produced by the EAF at 400 mg/kg dose, which reached 80.37%, followed by the hydromethanolic crude extract at the same dose level (74.01%) and then AF (55.24%). The n-hexane fraction showed the lowest ADI value (36.49%) at the same dose level. The finding showed higher anti-diarrheal activity by the standard drugs, then following the standard drugs the EAF and crude extract produce higher anti-diarrheal activity at their higher dose; however it is not superior to the standard drugs (such as atropine and loperamide) (Alemu et al., 2022). Similar findings have been reported by other studies, where the highest dose produced the largest ADI value (Tadesse et al., 2017; Teferi et al., 2019; Zayede et al., 2020).

**TABLE 5:** *In vivo* antidiarrheal index of the 80% hydromethanolic crude extract and solvent fractions of *Terminalia brownii* Fresen leaves.

Extract	Dose mg/kg	Dfreq (%)	Gmeq (%)	Pfreq (%)	ADI (%)
80% MeOH-E	NC	—	—	—	—
	PC	129.01	70.47	75.43	88.18
	MeOH 100	26.62	37.16	42.1	34.66
	MeOH 200	106.14	49.20	50.87	64.28
	MeOH 400	117.74	53.06	64.91	74.01
Solvent fraction	NC	—	—	—	—
	PC	148.62	67.65	72.7	90.07
	NHF 100	20	4.96	19.97	12.55
	NHF 200	30.58	19.18	23.58	24.00
	NHF 400	36.86	31.55	41.81	36.49
	NC	—	—	—	—
	PC	148.62	67.56	72.7	90.03
	EAF 100	44.30	46.13	30.89	39.81
	EAF 200	109.01	53.25	50.87	66.59
	EAF 400	145.88	59.29	60.04	80.37
	NC	—	—	—	—
	PC	152.52	68.07	75.02	92.00
	AF 100	26.43	14.81	24.97	21.38
	AF 200	57.23	26.72	48.23	41.93
	AF 400	99.63	30.55	55.41	55.24

MeOH, 80% methanol extract; NHF, n-hexane fraction; EAF, ethyl acetate fraction; AF, aqueous fraction; ADI, *in vivo* antidiarrheal index; Dfreq, delay in diarrhea onset (in % of control). Gmeq is the intestinal meal travel reduction (in % of control). Pfreq is the purging frequency as the number of wet stools reduce (in % of control); PC, positive control (loperamide or atropine); NC, negative control; administered with 10 mL/kg distilled water (for the crude extract and aqueous fraction) and 10 mL/kg of 2% Tween 80 (for n-hexane fraction and ethyl acetate fraction).

## Conclusion

According to the results obtained from the current study, the hydromethanolic crude extract and solvent fractions of *Terminalia brownii* Fresen leaves exhibit antidiarrheal activity, possibly through antimotility effects and the inhibition of fluid secretion. The antidiarrheal effect of the plant extract might be due to the presence of secondary metabolites identified in the extract. The findings suggest that extraction using hydromethanol (through the cold maceration technique) and fractionation using ethyl acetate were found to be more effective compared to other solvents. As a result, the present findings provide scientific evidence for the traditional use of the plant in the treatment of diarrhea.

## Data Availability

The raw data supporting the conclusions of this article will be made available by the authors, without undue reservation.
